# Effect of an innovative community based health program on maternal health service utilization in north and south central Ethiopia: a community based cross sectional study

**DOI:** 10.1186/1742-4755-11-28

**Published:** 2014-04-04

**Authors:** Mesganaw Fantahun Afework, Kesteberhan Admassu, Alemayehu Mekonnen, Seifu Hagos, Meselech Asegid, Saifuddin Ahmed

**Affiliations:** 1 Department of Reproductive Health and Health Service Management, School of Public Health, College of Health Sciences, Addis Ababa University, Addis Ababa, Ethiopia; 2 Federal Ministry of Health, Addis Ababa, Ethiopia; 3 Bill & Melinda Gates Institute for Population and Reproductive Health, Population, Family and Reproductive Health Johns Hopkins Bloomberg School of Public health, Baltimore, USA

**Keywords:** Skilled attendance at birth, Community based health programs, Health facility delivery, Antenatal care, Health Extension Worker, Ethiopia

## Abstract

**Background:**

Among Millennium Development Goals, achieving the fifth goal (MDG-5) of reducing maternal mortality poses the greatest challenge in Sub-Saharan Africa. Ethiopia has one of the highest maternal mortality ratios in the world with unacceptably low maternal health service utilization. The Government of Ethiopia introduced an innovative community-based intervention as a national strategy under the Health Sector Development Program. This new approach, known as the Health Extension Program, aims to improve access to and equity in essential health services through community based Health Extension Workers.

**Objective:**

The objective of the study is to assess the role of Health Extension Workers in improving women’s utilization of antenatal care, delivery at health facility and postnatal care services.

**Methods:**

A cross sectional household survey was conducted in early 2012 in two districts of northern and south central parts of Ethiopia. Data were collected from 4949 women who had delivered in the two years preceding the survey. Logistic regression analysis was performed to determine the association between visit by Health Extension Workers during pregnancy and use of maternal health services, controlling for the effect of other confounding factors.

**Results:**

The non–adjusted analysis showed that antenatal care attendance at least four times during pregnancy was significantly associated with visit by Health Extension Workers [Odds Ratio 3.46(95% CI 3.07,3.91)], whereas health facility delivery (skilled attendance at birth) was not significantly associated with visit by Health Extension Workers during pregnancy [Odds Ratio 0.87(95% CI 0.25,2.96)]. When adjusted for other factors the association of HEWs visit during pregnancy was weaker for antenatal care attendance [Adjusted Odds Ratio: 1.35(95% CI: 1.05, 1.72)] but positively and significantly associated with health facility delivery [Adjusted Odds Ratio 1.96(1.25,3.06)].

**Conclusion:**

In general HEWs visit during pregnancy improved utilization of maternal health services. Health facility delivery is heavily affected by other factors. Meaningful improvement in skilled attendance at birth (health facility delivery) should include addressing other factors on top of visits by HEWs during pregnancy and specific target oriented interventions during visits by HEWs to support skilled attendance at birth.

## Background

Among Millennium Development Goals (MDGs), achieving the goal for MDG5 (Maternal Health Goal), poses the greatest challenge in Sub-Saharan Africa [1-3]. Ethiopia has one of the highest maternal mortality ratios in the world [2-4] and maternal health service utilization in Ethiopia is low. The 2011 Ethiopian Demographic and Health Survey (EDHS 2011) reported that 34% of women used antenatal care (ANC), 10% women delivered with skilled attendance at birth (SAB) and 9% received postnatal care with regional variations [4].

To ameliorate this unacceptably low maternal care utilization, the Government of Ethiopia introduced an innovative community-based intervention as a part of the Health Sector Development Program II. The main aim of this new approach, known as the Health Extension Program (HEP), is to improve access to and equity in essential health care through community based outreach health services at the doorsteps of the residents [5-7]. The HEP was started in 2003 and services are provided by a new cadre of female health workers, known as Health Extension Workers (HEWs). Currently, more than 35000 HEWs are working in the country. Two HEWs are deployed in each k*ebele. Kebele* is the smallest administrative unit in Ethiopia. HEWs are full time employees of national health system, and are trained for one year after completing grade 10 of school. They are considered as the first point of contact of the community with the health system, delivering integrated preventive, promotive and curative health services, with a special focus on maternal and child health [6]. While they may provide basic ANC and may conduct home delivery, they may not be adequately trained in midwifery skills to proficiency.

Previous studies showed that the health extension program has contributed in improving contraceptive use [8] and utilization of some components of maternal health services. However, the evidence on improving skilled attendance at birth or health facility delivery by HEW visits is still limited. Two studies conducted between 2008–2010 [9,10] found no association of HEW visits with SAB, which is considered a key process indicator for MDG 5. These studies also reported poor knowledge of HEWs on maternal complications and maternal counseling.

The Health Sector Development Program IV (HSDP IV 2010/11–2014/15) set new and higher targets to increase deliveries attended by skilled attendants from 18% to 60%, and to decrease the maternal mortality ratio from 590 per 100,000 live births to 267 per 100000 livebirths [5]. Subsequently, several actions were taken to improve knowledge and skills of HEWs including the integrated refresher training of HEWs [11,12]. The current study will therefore provide information on the effects of HEWs visit on maternal care utilization in recent period.

## Methods

A community based cross sectional study was conducted in two of the nine administrative regions of the Ethiopia—Tigray in the north; and the Southern Nations, Nationalities, and People’s Region (SNNPR) in the south. These study areas were selected purposefully. These two regions are home to a university that is currently being mentored by Addis Ababa University (AAU) and run a health and demographic surveillance system (HDSS) in the selected for the study districts. Maternal health service utilization indicators in the selected regions indicate Tigray has ANC at least once levels of 65%, SAB (11.6%), and post natal visits in the first 2 days. SNNPR had ANC at least once rate of 41%, SAB (6.2%), and postnatal visit in the first 2 days (5.5%) [4].

We selected 12 *kebeles* from the two districts namely Wukro in northern Ethiopia and Butajira in south central Ethiopia. Six *kebeles* each were selected using simple random sampling procedure from HDSS sites and non-HDSS sites in each district to control the effect exposure to health and demographic surveillance activities. These 12 *Kebeles* were selected proportionally from urban and rural areas, 2 from urban *kebeles* and 10 from rural *kebeles.*

The study population included all women 15–49 of age, married or unmarried, who delivered within last two years in the selected *kebeles,* irrespective of the status of birth outcome whether live birth or stillbirth.

The Ethiopian Crude Birth Rate (CBR) was estimated at 34.5/1000 midyear population [4]. With this CBR on an average we expected to have about 104 births per year in a *kebele* with an approximate population size of 3000. In 12 *kebeles* in each of the two districts, about 2496 deliveries were expected per site during 2 years of retrospective observation period. Thus, we expected about 4992 women as the target population in these study areas for this and other studies which assessed differentials in health service utilization by different determinants.

Of the 4981 women approached for the interview 14 women were not available for interview after repeat visits and 18 questionnaires were discarded because of inconsistencies. A total of 4949 (99, 3%) women were finally included in the study.

Home visit by a HEW at least once during pregnancy was taken as the independent variable of interest and ANC visit at least four times and skilled attendance at birth (health facility delivery) as the dependent variable.

Initial sample size calculations for this and other studies on maternal health service utilization were based on SAB rate of 16% around the time of the survey [8]. With the sample of women included in the study a difference of 4% would be detected between those who were visited by HEWs at least once and those not visited with a power of 80% and a maximum design effect of 2. Ethical approval for this study was obtained from the Institutional Review Board Office, John Hopkins Bloomberg School of Public Health and Institutional Review Board of the College of Health Sciences, Addis Ababa University.

### Data collection

Data collection was conducted by twenty trained and experienced female interviewers, who were high school graduates using questionnaire that contained socio-demographic characters tics of the respondents, visit by HEWs during pregnancy and use of maternal health services. Data collection activities were monitored by two supervisors in each study district. The supervisors had a minimum of a bachelor degree education and previous experience in supervising community based data collection.

A sampling list of household members was constructed through a census of households and eligible women who had delivered during the previous two years were identified. All eligible women who voluntarily consented participated in the study after listening to the interviewer reading the informed consent. Supervisors randomly interviewed about 4% of the women for checking the reliability of responses as a part of data quality monitoring. A pretest was conducted in a district not selected for the study and some revisions were made on the questionnaire to improve clarity and understandability by the respondents.

### Data entry and analysis

Data were double entered in a customized data entry program by experienced data clerks. Data analysis was performed using STATA 12 (Stata Corp, Texas). Data quality was checked by examining missing responses, inappropriate values, and violation of skip rules.

A wealth index score was constructed for each household with a principal component analysis of household durable goods, household structure conditions (eg, materials used to construct wall, roof, floor of houses, type of toilets), and land possessions. Households were ranked according to the total wealth score and then divided into wealth quintiles as a proxy of household socio-economic status.

We examined the distribution of socio-demographic characteristics of the study population, the coverage of maternal health services and association between visit by HEWs and other factors with use of maternal health services (Antenatal Care at least four times, Institutional Delivery including health posts, health centers and hospitals and Postnatal Care within three days). Multi collinearity was checked by calculating variance inflation factor (VIF) and we applied complex survey data analysis specifying survey design and sampling unit (*kebeles*). The variance was adjusted with Taylor linearized variance estimation method. Multivariate logistic regression analysis adjusted for cluster level sampling *(kebele)* was then run to control for the effect of other factors for which literature review showed association with maternal health service utilization (Eg 13.). Odds ratios (95% confidence intervals) were calculated to determine the association between antenatal care attendance at least four times, institutional delivery, postnatal care within three days of delivery and predictor variables.

## Results

Visit of HEWs at least once by socio-demographic characteristic of the study population is shown in Table 1. About 65% of the women in rural areas and 53% in urban areas answered that they were visited by HEWs during pregnancy of the index child. Two thousand forty six (67%) of those who were unable to read and write were visited by HEWs, whereas it was about 38% of those who had college education that were visited by HEWs. HEW’s visit during pregnancy was reportedly lower for Muslims and Protestants (28% each) compared by Orthodox Christians. About 90% and 36% of the housewives and the traders or employees respectively answered that they were visited by HEWs during pregnancy of the index child.

**Table 1:** Socio-demographic characteristics of women who delivered a baby in the two years preceding the survey by HEWs visit during pregnancy in north and south central Ethiopia, 2012

	**Visit by HEWs during pregnancy at least once**	
**Characteristics**	**Yes (n,%)**	**No (n,%)**
**Place of residence**		
Urban	620(53.0)	550(47.0)
Rural	2465(65.2)	1314(34.8)
**Level of education**		
None	2046(66.0)	1052(34.0)
Primary	757(55.5)	608(44.5)
Secondary	252(62.7)	150(37.3)
College	30(35.7)	54(64.3)
**Marital status**		
Currently married	2921(62.1)	1785(37.9)
Widowed, divorced, never married	164(67.5)	79(32.5)
**Religion**		
Orthodox Christian	2602(79.3)	679(20.7)
Muslim	379(26.8)	1033(73.2)
Protestant	529(26.8)	142(63.2)
Catholic	49(83.1)	10(17.0)
**Occupation**		
Housewife	1754(89.9)	197(10.1)
Housewife and farm work	992(46.8)	1127(53.2)
Trade and other employee	217(36.4)	379(63.6)
Others	122(43.1)	161(56.9)
**Number of pregnancies**		
1	522(54.4)	437(45.6)
2-4	1390(62.6)	832(37.4)
5-6	680(62.3)	346(33.7)
7 and above	4939(66.4)	249(33.6)
**Number of deliveries**		
1-2	1049(59.1)	726(40.9)
3-4	858(61.2)	543(38.8)
5-6	680(62.3)	346(33.7)
7+	493(66.4)	249(33.6)

Table 2 shows the maternal health service utilization status of women who delivered in the previous two years. Four thousand four hundred eight (89.1%) women reported that they had attended ANC at least once, whereas 57.6% had attended ANC at least four times during the pregnancy of the index child. About 29% of the women started to attend ANC during the first trimester whereas 68.3% started attending ANC during the second trimester. Regarding place of delivery, three-fourth of the women delivered the index child at home, followed by delivery in government hospitals (16.1%) and health centers (7.8%). About 88% said that they had visited a health facility for post natal care. Of these 3.7% visited a health facility within 24 hours and 10.2% within 3 days after delivery.

**Table 2:** Maternal health services utilization among women who delivered in the two years preceding the survey in north and south central Ethiopia, 2012

**Maternal health services**	**Frequency**	**Percent**
**Had antenatal care at least once**		
Yes	4408	89.1
No	541	10.9
**Time at first ANC visit**		
First trimester	1255	28.5
Second trimester	3003	68.3
Third trimester	144	32.7
N = 4402		
**Had antenatal care at least four times**		
Yes	2850	57.6
No	2099	42.4
**Place of ANC attendance**		
Health post	2001	45.6
Health center	1748	39.8
Hospital	639	14.6
n = 4388		
**Place of delivery**		
Home	3700	74.9
Health post	34	0.7
Health center	386	7.8
Government hospital	793	16.1
Private hospital	24	0.5
**Had post natal care**		
Yes	4343	88.1
No	589	11.9
**Time at post natal care visit**		
Within 24 hours	162	3.7
Within 3 days	444	10.2
Within 7 days	2061	47.5
45 days after delivery	886	20.4
Later	483	11.1
Unspecified	307	7.1
**Place of PNC visit**		
Health post	2500	57.9
Health center	1123	26.0
Government hospital	648	15.0
Private clinic	24	0.6
Private hospital	20	0.5
Unspecified	2	0.05

Table 3 shows the association of visit by HEWs during pregnancy and other factors with ANC attendance at least four times. The odds of attending ANC at least 4 times by those who were visited during pregnancy at least once was about 3 times higher than those who were not visited by HEWs, adjusted for other factors. Rural residents had about 60% chance of attending ANC at least four times compared to urban residents. Other factors that showed significant association with attendance at least four times during pregnancy include wealth quintile and number of pregnancies.

**Table 3:** Association of HEWs and other factors with with ANC at least four times in north and south central Ethiopia, 2012

**Characteristics**	**Had ANC four times**		**Crude OR (95% CI)**	**Adjusted OR (95% CI)**
	**YES (n,%)**	**NO (n,%)**		
**Visit by HEWs during pregnancy**				
None	726(38.9)	1138(61.1)	1.00	1.00
Visisted at least once	2124(68.8)	961(31.2)	3.46(2.11,5.66)	3.42(2.62,4.47)*
**Place of residence**				
Urban	796(68.0)	374(32.0)	1.0	1.00
Rural	2054(54.4)	1725(45.6)	0.56(0.24,1.29)	0.66(0.49,0.89)*
**Mothers age (years)**				
15-19	126(56.3)	98(43.7)	1.0	1.00
20-29	1474(58.3)	1053(41.7)	1.09(.088,1.35)	1.07(0.79,1.46)
30-39	1088(57.3)	810(42.7)	1.04(0.77,1.42)	1.19(0.78,1.82)
40-49	160(55.0)	131(45.0)	0.95(0.58,1.56)	1.40(0.73,2.69)
**Women’s education**				
None	1736(56.1)	1362(43.9)	1.0	1.00
Primary	764(56.0)	601(44.0)	0.99(0.81,1.23)	0.95(0.81,1.12)
High school	290(72.1)	112(27.9)	2.03(1.20,3.43)	1.29(1.06,1.59)*
College/University	60(71.4)	24(28.6)	1.96(0.82,4.72)	1.35(0.75,2.42)
**Marital status**				
Currently married	2717(57.7)	1989(42.3)	1.0	1.00
Currently unmarried	133(54.7)	110(45.3)	0.89(0.62,1.27)	0.72(0.51,1.02)
**Wealth quintile**				
Poorest	718(72.2)	277(27.8)	1.0	1.00
Poor	599(60.3)	395(39.7)	0.59(0.33,1.03)	0.67(0.42,1.08)
Middle	408(41.2)	582(58.8)	0.27(0.16,0.46)	0.43(0.26,0.69)*
Rich	456(46.7)	521(53.3)	0.34(0.21,0.54)	0.52(0.33,0.83)*
Richest	669(67.4)	324(32.6)	0.79(0.42,1.56)	0.883(0.58,1.34)
**Number of pregnancies**				
1	601(62.7)	358(37.3)	1.0	1.00
2-4	1320(59.4)	902(40.6)	0.87(0.67,1.14)	0.84(0.65,1.08)
5-6	572(55.8)	454(44.2)	0.75(0.51,1.11)	0.63(0.41,0.97)*
7 and above	357(48.1)	385(51.9)	0.55(0.38,0.81)	0.43 (0.30, 0.62)*

*p < 0.05.

Visit by HEWs was not significantly associated with institutional delivery in crude analysis. Adjusted for other factors the odds of delivering the index child in health facility was about twice among those who were visited by HEWs compared with those who were not. Mother’s age, educational status, place of residence, wealth quintile and number of pregnancies were also significantly associated with place of delivery in multivariate analysis (Table 4).

**Table 4:** Association of HEWs visit during pregnancy and other factors with place of delivery in north and south cetral, Ethiopia 2012

**Characteristics**	**Place of delivery**		**Crude OR (95% CI)**	**Adjusted OR (95% CI)**
	**Health facility (n,%)**	**Home (n,%)**		
**Visit by HEWs during pregnancy**				
None	497(26.7)	1367(73.3)	1.00	1.00
Visisted at least once	740(24.0)	2345(76.0)	0.87(0.25,2.96)	1.96(1.25,3.06)*
**Place of residence**				
Urban	704(60.2)	466(39.8)	1.00	1.00
Rural	533(14.1)		0.11(0.03,0.30)	0.49(0.26,0.90)*
**Mothers age (years)**				
15-19	80(35.7)	144(63.3)	1.00	1.00
20-29	714(28.3)	1813(71.8)	0.71(0.55,0.91)	1.25(0.82,1.90)
30-39	408(21.5)	1490(78.5)	0.49(0.32,0.76)	2.25(1.32,3.85)*
40-49	32(11.0)	259(89.0)	0.22(0.14,0.35)	2.35(1.27,4.33)*
**Women’s education**				
None	440(14.2)	2658(85.8)	1.00	1.00
Primary	434(31.8)	931(68.2)	2.81(2.25,3.52)	1.19(0.97,1.46)
High school	291(72.4)	111(27.6)	15.84(8.76,28.65)	2.757(1.90,3.99)*
College/University	72(85.7)	12(14.3)	36.25(15.40,85.30)	3.943(1,64,9.46)*
**Marital status**				
Currently married	1144(24.3)	3562(75.7)	1.00	1.00
Currently unmarried	93(38.3)	150(61.7)	1.93(1.19,3.12)	1.317(0.94,1.84)
**Wealth quintile**				
Poorest	79(7.9)	916(92.1)	1.00	1.00
Poor	122(12.3)	872(87.3)	1.62(1.09,2.41)	1.61(1.15,2.24)*
Middle	85(8.6)	905(91.4)	1.09(0.64,1.59)	1.37(0.87,2.15)
Rich	231(23.6)	746(76.4)	3.60(2.10,6.13)	3.73(2.34,5.95)*
Richest	720(72.5)	273(27.5)	30.60(15.22,61.43)	16.33(7.59,35.11)*
**Number of pregnancies**				
1	474(49.4)	485(50.6)	1.00	1.00
2-4	532(23.9)	1690(76.1)	0.32(0.25,0.41)	0.35(0.26,0.46)*
5-6	158(15.4)	868(84.6)	0.87(0.13,0.26)	0.24(0.16,0.35)*
7 and above	73(9.8)	669(90.2)	0.11(0.07,0.17)	0.18(0.12,0.26)*

*p < 0.05.

On the other hand those who were visited by HEWs during pregnancy were highly likely to attend post natal care during the first three days [AOR 3.68(2.05,6.59)] while other factors except number of pregnancies did not appear to make a significant difference in postnatal care visit within the first three days after delivery (Table 5).

**Table 5:** Association of HEW’s visit during pregancy and other factors with postnatal care service within three days in north and south central Ethiopia, 2012

**Characteristics**	**Postnatal care service within three days**		**Crude OR (95% CI)**	**Adjusted OR (95% CI)**
	**YES (n,%)**	**NO (n,%)**		
**Visit by HEWs during pregnancy**				
None	91(4.9)	1173(95.1)	1.0	1.0
Visited	515(16.7)	2570(83.3)	3.90(2.42,6.29)	3.68(2.05,6.59)*
**Place of residence**				
Urban	174(14.9)	996(85.1)	1.0	1.0
Rural	432(11.4)	3347(88.6)	0.74(0.21,2.64)	0.85(0.57,1.28)
**Mothers age (years)**				
15–19	23(10.3)	201(89.7)	1.0	1.0
20–29	302(11.9)	2225(88.1)	1.19(0.69,2.05)	1.33(0.70,2.53)
30-39	246(13.0)	1652(87.0)	1.30(0.75,2.27)	1.71(0.86,3.44)
40-49	33(11.3)	258(88.7)	1.12(0.57,2.18)	1.74(0.78,3.85)
**Women’s education**				
None	345(11.3)	2747(88.7)	1.0	1.0
Primary	161(11.8)	1204(88.2)	1.05(0.76,1.44)	1.13(0.96,1.32)
High school	81(20.1)	321(79.9)	1.97(1.04,3.74)	1.69(1.06,2.69)*
College/University	13(15.5)	71(84.5)	1.43(0.91,2.26)	1.56(0.38,6.35)
**Marital status**				
Currently married	556(11.8)	4150(88.2)	1.0	1.0
Currently unmarried	50(20.6)	193(79.4)	1.93(1.23,2.88)	1.67(1.05,2.65)*
**Wealth quintile**				
Poorest	169(17.0)	826(83.0)	1.0	1.0
Poor	131(13.2)	863(86.8)	0.74(0.49,1.11)	0.80(0.58,1.11)
Middle	7397.4)	917(92.6)	0.39(0.21,0.71)	0.58(0.34,0.98)*
Rich	88(9.0)	889(91.0)	0.48(0.25,0.91)	0.65(0.37,1.16)
Richest	145(14.6)	848(85.4)	0.83(0.24,2.93)	0.85(0.36,2.01)
**Number of pregnancies**				
1	145(15.1)	814(84.9)	1.0	1.0
2-4	271(12.2)	1951(87.8)	0.78(0.60,1.02)	0.76(0.5,1.02)
5-6	114(11.1)	912(88.9)	0.70(0.48,1.02)	0.59(0.38,0.94)*
7 and above	76(10.2)	666(89.8)	0.64(0.41,1.00)	0.53(0.29,0.93)*

*p < 0.05.

## Discussion

Visit by HEWs during pregnancy improved ANC utilization. This is expected as one of the main reasons for the HEWs home visit is to encourage women to attend ANC. A study mentioned above [8] reported higher coverage of ANC where visits of HEWs and voluntary community health workers were frequent.

Visit by HEWs during pregnancy was not statistically significantly associated with institutional delivery in crude analysis. However, adjusted for other factors, visit by HEWs during pregnancy had a positive and significant effect on institutional delivery. This may be the result of the overwhelming effect of other factors. Relevant literature shows the mechanism of how different factors affect maternal health services [13]. For example urban residents have better access to health facilities both in number, type and level of care they provide in this and other developing country contexts, which may encourage the use of facility delivery by the residents.

As in the case of ANC at least four times those who were visited by HEWs during pregnancy were highly likely to attend post natal care during the first three days. The reason for the similarity between the influence of .visit by HEWs on ANC and postnatal care attendance, and the difference from health facility delivery may need to be explored further. However, it may be speculated that ANC and postnatal care visits can be planned, where as labor is often unplanned and delivery in health facilities needs preparedness in terms of resources and decision making. This may also explain the observed difference between ANC attendance and skilled attendance at birth (health facility delivery) rates in this and other studies [4,8]. Interviews with 19 HEWs in the study area revealed that only 12 of the HEWs informed women about “*Birth Preparedness*”, *(Unpublished report from this research*) the main component of which is planning and preparing for a skilled attendant at birth. Several studies have documented the importance of birth preparedness in seeking assistance of skilled birth attendants [14,15]. An earlier study in Tigray administrative region of Ethiopia reported low rate of birth preparedness [16].

There is an agreement about the relationship of HEWs visit and use of ANC services with studies conducted in Ethiopia earlier [9,10]. In a way, the findings of the effect of HEWs visit on health facility delivery (skilled attendance at birth) are also similar with the previous studies because no significant association was observed between visit by HEWs and health facility delivery in crude analysis. However, adjusted for other factors visit by HEWs during pregnancy was significantly associated with health facility delivery in this study thus revealing the fact that not much improvement can be expected in skilled attendance at birth by the visits of HEWs alone, without attending to the effects of other factors. Policies and strategies to improve skilled attendance at birth should consider these findings into consideration.

Four thousand four hundred eight women (89%) reported to have attended ANC at least once and 2850 (57%) at least four times. This is much higher than reported by the Ethiopian Demographic Health Survey which reported (43%) [4]. While this may be related to a general improvement of the ANC utilization after the data collection of EDHS 2011, the reasons may need to be explored further.

The majority of the women (68.3%) started attending ANC during the second trimester which is similar to the findings of the EDHS 2011. It appears that the health benefits of early ANC attendance for the mother and fetus (newborn) are not lost by the study population. Thus efforts are required to mobilize women for ANC as early as possible.

About 25% of the women delivered in health facilities. Although far from optimal, this appears to be higher than what was reported (10%) in the recent EDHS [4] and a report of the Federal Ministry of Health [12]. Quite a high proportion (87.7%) of women said that they had visited a health facility for postnatal care. Those who reported to have had PNC before 45 days were 3594 (71.8%). The EDHS 2011 [4] reported that 91.5% of women did not receive postnatal care within 41 days after delivery and it was 1% who received post natal care within 1–2 days. Thus there is a large difference in the reported use of postnatal care service in this study compared to the EDHS 2011 [4]. The finding of this study is also higher than the findings of an earlier study in four major administrative regions of the country [8].

The same argument as that of increased ANC attendance can be made for increased skilled attendance at birth and postnatal care in this study. There may have been more concerted efforts to improve maternal health services utilization following the unacceptably low coverage reported by the EDHS 2011 [4].

On the other hand, reports about use of postnatal care can be influenced by the purpose and timing of visit. In some instances postnatal care coverage calculations consider visits for immunization of children, child sickness and accompanying family members, while in others this may be restricted to maternal health check up. This may affect postnatal care coverage leading to inflations [17].

### Limitations of the study

This study assessed the effect of HEWs visit on maternal health utilization in two districts that represented urban and rural populations. The study areas were purposively selected. The study areas having health and demographic surveillance system may lead to the study population having better awareness and health seeking behaviors. Thus generalizations to the overall Ethiopian diverse population should be made cautiously. In addition, the cross sectional nature of the study has inherent limitations for establishing cause and effect relationships. As an observational study, it may also not have considered all potential confounding factors.

## Conclusions

HEWs visit during pregnancy has generally improved utilization of maternal health services. Health facility delivery is heavily affected by other factors and possibly due to inadequate performance of HEWs to promote health facility delivery during ANC visits. Meaningful improvement in skilled attendance at birth (health facility delivery) should include other interventions on top of visits by HEWs during pregnancy. Improvement in the messages to be delivered during home visits and ANC care by HEWs should be clearly spelt out to inform the advantages of skilled attendance at birth and support preparedness for skilled attendance at birth (health facility delivery). In depth assessment of the factors affecting service utilization using qualitative methodology is recommended.

## Competing interests

The authors declare that they have no competing interests.

## Authors’ contributions

MFA initiated the research and drafted the proposal. KA contributed to the refinement of the research question. KA, AM, SH, MA and SA contributed to the proposal development. AM, SH, MA supervised and monitored the data collection process. SA led the process of data analysis. SA, MA, MFA, SH and AM participated in data cleaning and analysis. All authors provided in puts to the final manuscript, read and approved it.
